# Promoter Trapping in Microalgae Using the Antibiotic Paromomycin as Selective Agent 

**DOI:** 10.3390/md10122749

**Published:** 2012-12-04

**Authors:** Marta Vila, Encarnación Díaz-Santos, Marta de la, Herminia Rodríguez, Ángeles Vargas, Rosa León

**Affiliations:** 1 Biochemistry Laboratory, Experimental Sciences Faculty, University of Huelva, Huelva 27071, Spain; E-Mails: marta.vila@dqcm.uhu.es (M.V.); encarnacion.diaz@dqcm.uhu.es (E.D.-S.); marta.delavega@dqcm.uhu.es (M.V.); 2 Institute of Plant Biochemistry and Photosynthesis, CIC Cartuja, University of Seville and CSIC, Seville 41092, Spain; E-Mails: hrm@us.es (H.R.); avargas@us.es (A.V.)

**Keywords:** *APHVIII*, *Chlamydomonas reinhardtii*, microalgae transformation, paromomycin, promoter trapping

## Abstract

The lack of highly active endogenous promoters to drive the expression of transgenes is one of the main drawbacks to achieving efficient transformation of many microalgal species. Using the model chlorophyte *Chlamydomonas reinhardtii* and the paromomycin resistance *APHVIII* gene from *Streptomyces rimosus* as a marker, we have demonstrated that random insertion of the promoterless marker gene and subsequent isolation of the most robust transformants allows for the identification of novel strong promoter sequences in microalgae. Digestion of the genomic DNA with an enzyme that has a unique restriction site inside the marker gene and a high number of target sites in the genome of the microalga, followed by inverse PCR, allows for easy determination of the genomic region, which precedes the *APHVIII* marker gene. In most of the transformants analyzed, the marker gene is inserted in intragenic regions and its expression relies on its adequate insertion in frame with native genes. As an example, one of the new promoters identified was used to direct the expression of the *APHVIII *marker gene in *C. reinhardtii*, showing high transformation efficiencies.

## 1. Introduction

In recent years, the number of genetically modified microalgal species has been slowly increasing, but it is still scanty. The lack of specific promoters and good methods for DNA delivery inside the nucleus are the main drawbacks to achieving efficient transformation of many of these species. The first and best studied transformed microalga is *Chlamydomonas reinhardtii*, which is still the favorite model system. This freshwater chlorophyte was transformed in 1989 by the complementation of a mutant form of the *NIT1* gene with the corresponding homologous nitrate reductase gene [1]. The genetic transformation of the diatoms *Cyclotella criptica* and *Navicula saprophila *was first reported in 1995 [2] and soon thereafter reports describing the transformation protocols for *Phaeodactylum tricornutum* appeared [3]. Since then, a significant number of selectable markers, promoters and new procedures for efficient introduction of DNA into the *Chlamydomonas reinhardtii* and *Phaeodactylum tricornutum* nucleus have been developed [4]. Nevertheless, the number of transformed species has timidly increased, and the majority of the work on transgenic microalgae is still being performed with the model species, especially with the chlorophyte *Chlamydomonas reinhardtii*. In this system, easy procedures for genetic manipulation of nuclear and plastidic genomes and a complete kit of molecular and bioinformatic tools have been developed [5,6,7,8,9].

Heterologous promoters have allowed transient or low efficient expression of marker genes in some species of green microalgae [10,11]. However, at present, the best transformation efficiencies and the most stable transformants are obtained with endogenous promoters [12]. As concerns the classical microalgal transformation systems referred, *Chlamydomonas* and *Phaeodactylum*, there is a wide collection of strong constitutive homologous promoters, such as the rubisco small subunit *RBCS2* promoter fused to the heat shock protein (*HSP70A*) promoter of *C. reinhardtii *or the fucoxanthin chlorophyll binding protein (*FCPA*) promoter of *P. tricornutum*, which are extensively used to drive the expression of heterologous proteins in these microalgae [13]. However, the isolation of endogenous promoters for the genetic transformation of other chlorophytes has only been described in *Chlorella sorokiniana* [14], *Dunaliella* [15,16], *Haematococcus pluvialis *[17], *Ostreococcus tauri* [18] and *Nannochloropsis* [19,20]. In the three first cases, the transformation was based on complementation of deficient mutants with a homologous gene of nitrate reductase, for *Chorella sorokiniana* and *Dunaliella*, and of phytoene desaturase, for *Haematococcus pluvialis*. In some species, endogenous strong promoters, controlling genes that encode abundant proteins, did not yield good transformation efficiencies. Walker *et al. *[12] isolated the flanking regions of *Dunaliella tertiolecta RBCS2* genes. These promoters were shown to drive the expression of bleomycin resistance gene (*BLE*) in the related microalga *Chlamydomonas reinhardtii* [12] and in the own *Dunaliella tertiolecta*, but with very low efficiency [16]. This shows that much more work is necessary to develop transformation systems with endogenous constitutive promoters that allow an efficient and stable expression of transgenes in the wide range of microalgal strains. 

The choice of highly active endogenous promoters to drive the expression of transgenes is thus a critical first step in the development of efficient transformation systems in microalgae. New promoters are generally isolated by chromosomes walking based on gene encoding sequences or retrieved from the genome sequence data, if available. Promoter trapping method has also allowed the isolation and characterization of a good number of novel promoters in higher plants [21,22]. The method basically consists on the generation of a collection of transformants with random insertions of a promoter-less reporter gene, and the subsequent sequencing by different strategies of the genomic region preceding the marker insertion in the transformants selected. However, in microalgae only a few examples of promoter trapping have been reported [23,24].

A good number of marker/reported genes have been isolated for *Chlamydomonas reinhardtii*, some of which have been successfully used for screening of differentially regulated genes [25] and for identification of new genes by forward [26,27,28] or reverse [29] genetic approaches. In 1997, Haring and Beck [23] described the identification of new promoters in *Chlamydomonas* using the promoter-less radial spoke protein (RSP3) as selectable marker. However, this system involves the necessary use of a mutant strain as a host. In the present work, we propose the use of the promoter-less gene *APHVIII *from *Streptomyces rimosus*, which encodes for an aminoglycoside 3′-phosphotransferase and provides resistance to the antibiotic paromomycin, for promoter trapping in microalgae. The *APHVIII *gene has been shown to work under the control of endogenous promoters in *Chlamydomonas* [30] and in the closely related species *Gonium pectorale* [31]. In addition, we have shown that the antibiotic paromomycin can be used as selective agent in a good number of chlorophytes. In this work, we have fused the *APHVIII* gene with the 3′ UTR region of the nopaline synthase and we have used this construction for transformation of *Chlamydomonas reinhardtii*. We demonstrate that random insertion of the *APHVIII* promoterless gene and subsequent sequencing of the marker flanking region allows the easy and quick identification of new promoter sequences. The method is validated in the model chlorophyte *Chlamydomonas reinhardtii* and the possibility of using it on other microalgae is discussed.

## 2. Results

### 2.1. Construction of a Promoter-Less Cassette Suitable for Promoter Trapping in Microalgae

**Figure 1:**
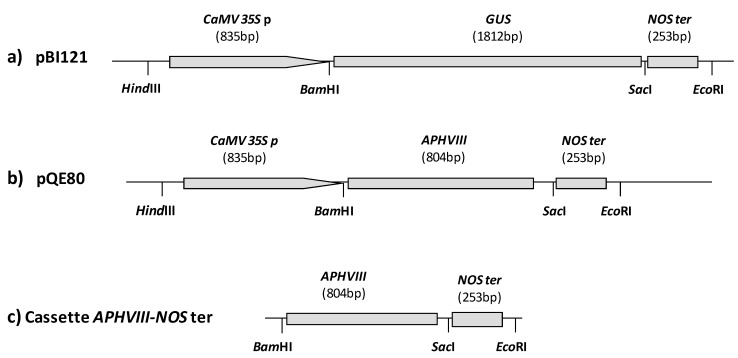
Schematic structure the *APHVIII-NOS *ter promoter-less cassette and the constructions generated during its construction. The *CaMV 35S *p*-GUS-NOS *ter cassette was excised from the binary vector pBI121 (**a**); The *GUS* gene was substituted by *APHVIII *(**b**) and finally the *APHVIII-NOS *ter promoter-less cassette excised by digestion (**c**). The more relevant restriction sites are indicated.

To synthesize the *APHVIII *promoter-less construction, the *CaMV 35S *p*-GUS-NOS *ter cassette was excised from the binary vector pBI121 [32], and subcloned between the *Hind*III and *Eco*RI sites of pQE80 plasmid (Qiagen). The cassette, which contains the *35S* promoter of the Cauliflower mosaic virus, the β-glucuronidase (*GUS*) gene and the *NOS* terminator region from *Agrobacterium tumefaciens*, was modified by exchanging the *GUS* gene by the A*PHVIII *gene from *Streptomyces rimosus*, previously obtained by PCR amplification from the pSI103 plasmid [33]. The resulting construction contains the *APHVIII *gene flanked by the *CaMV 35S* promoter and the *NOS* terminator region. The *APHVIII *promoter-less cassette was obtained from this construction by digestion with *Bam*HI and *Eco*RI (Figure 1).

The reporter gene chosen, *APHVIII*, has proved to be a good marker gene for *Chlamydomonas* [30], and other chlorophytes. It has very stable activity and provides an efficient screening system. Here we carry out the molecular characterization of the transformants obtained with the *APHVIII* promoter-less construction to check if the expression of the marker gene is the result of *in vivo* fusion with endogenous promoters. The *APHVIII* gene is simultaneously used as selectable and reporter gene. The 3′ UTR of *RBCS2*, which has been reported to have a certain antisense promoter activity [30], was avoided as terminator region; instead, we used the nopaline synthase terminator region. In this way, we ensure that the paromomycin tolerance phenotype in the obtained mutants is due to endogenous promoters and not to the reverse promoter activity of 3′ *RBCS2 *UTR. 

### 2.2. Transformation of *C. reinhardtii* with the *APHVIII *Promoter Trapping Construction and Analysis of Transformants

*C. reinhardtii* was transformed with 150 ng of the *APHVIII*-*NOS *ter cassette excised by digestion with *Bam*HI and *Eco*RI restriction enzymes from the pQE80-*CaMV 35S *p-*APHVIII*-*NOS* ter plasmid (Figure 1). A control with an equivalent quantity of the linearized pSI103 plasmid was also included. This plasmid designed by Sizova and coworkers [30] contains the *APHVIII* gene flanked by the heat shock protein 70A (*HSP70A*), the rubisco small subunit 2 (*RBCS2*) promoters and the 3′ untranslated region of the *RBCS2*. The transformation rate observed in *Chlamydomonas reinhardtii* transformed with the linearized pSI103 control is 1.8 × 10^−6^ transformants per cell and µg of DNA while the transformation with the promoter-less cassette was 2.6 × 10^−7^, which means about 14% of the transformation rate observed for the control plasmid (Table 1).

Twenty transformation reactions with 150 ng of the trapping cassette allowed the isolation of 70 paromomycin-resistant transformants that were cultured in TAP medium with increasing quantities of paromomycin over three days. 100 µL of a well-grown culture of each mutant were inoculated in 3 mL tubes of TAP medium, containing 30, 40 and 50 µg mL^−1^ of paromomycin. Five per cent of the transformants lost their resistance to the antibiotic and were discarded; about 24 transformants (35%) were able to survive in the presence of 50 µg mL^−1^ of paromomycin. Ten transformants were selected among those that grew more vigorously in the presence of paromomycin (50 µg mL^−1^) for further molecular analysis. 

**Table 1:** Comparison between the efficiency of transformations with the promoter-less *APHVIII-NOS* cassette and the control pSI103 plasmid. The number of transformants obtained per reaction (mean value and error) and the transformation rate are shown for the linearized pSI103 plasmid and for the *APHVIII-NOS ter* cassette. About 150 ng of DNA and 10^8^ cells were used for each transformation.

Transformation cassette	*N* of transformants per reaction	Transformation rate (transformants cell^−1^ μg^−1^ DNA)
Linearized pSI103	28 ± 5 (*n* = 3)	1.8 × 10^−6^
*APHVIII-NOS* ter cassette	4 ± 2 (*n* = 20)	2.6 × 10^−7^

### 2.3. Molecular Characterization of the Selected Transformants

DNA from the obtained transformants was isolated by rapid DNA isolation method as described in the experimental section to check integration of the marker gene in the genome. A simple Real-Time qPCR based method, previously validated by Gonzalez-Ballester *et al.* [29] and based on *NIA1* (nitrate reductase) as a unique-copy reference gene, was used to determine the *APHVIII* copy number in each transformant. Briefly, the method consists on comparing the Ct value for *APHVIII* relative to the Ct for amplification of *NIA1* in each transformant and in a selected control transformant with a unique copy of the *APHVIII *gene.

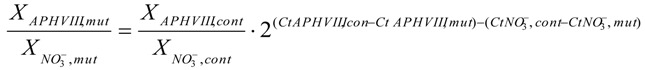
(1)


We found that practically all of the mutants analyzed (80%) had a single copy of the tag. The use of small quantities of DNA in the transformation experiments has been described to avoid or minimize multiple insertions [29].

**Figure 2:**
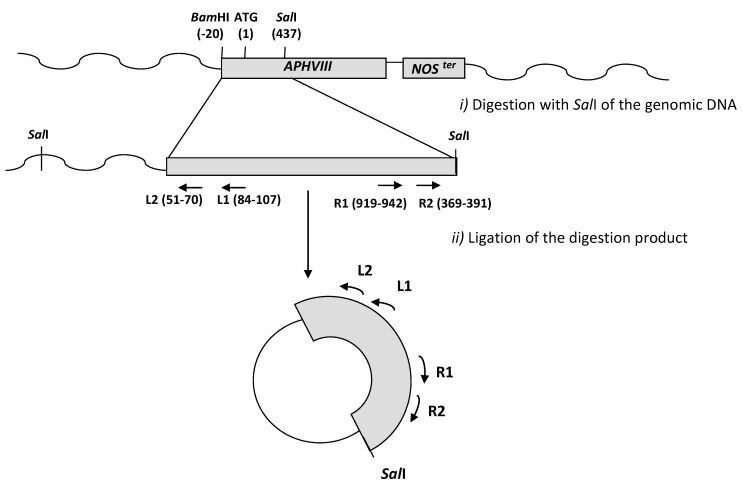
Scheme of the inverse PCR strategy used for amplification of the regions preceding the *APHVIII *marker insertion. The approximate sites for primers hybridation and restriction enzymes digestion are indicated.

The putative endogenous promoter region responsible for the expression of the *APHVIII* was identified amplifying by inverse PCR and sequencing the region upstream the marker gene, in seven of the paromomycin resistant transformants selected. All the selected transformants showed resistance to high concentrations of the antibiotic paromomycin (50 µg mL^−1^) and had a single marker gene insertion. The genomic DNA located upstream the marker gene was isolated by an inverse nested PCR and sequenced, as described in materials and methods using primers ParaL2 and ParaR2 (Figure 2). 

In all cases, the sequence obtained by amplification through inverse PCR included a short fragment homologous to the *APHVIII* gene from *Streptomyces rimosus*, which confirmed specificity, and a fragment that showed high identity with the genome of *C. reinhardtii*. These fragments were compared with the last version of the *C. reinhardtii *genome database at the joint genome institute (DOE-JGI) [34] by the BLAST tool. The insertion site and the protein encoded by the interrupted gene, when available, are shown in Table 2. 

We found that in all the transformants analyzed the insertion took place in intragenic regions, preferably inside the protein encoding region. Only in two of the analyzed transformants (1–14 and 4–15) the marker gene was inserted within the promoter region. In these two cases, the sense of the *APHVIII* insertion is reverse to the sense of the coding region directed by the interrupted promoter (Table 2), suggesting that the promoter regions in transformants 1–14 and 4–15 could have an antisense promoter activity.

We have found a variety of functions for the proteins encoded by the genes localized in the insertion sites of the analyzed transformants. For example, transformant 4–5 showed an insertion in the last intron of the gene encoding for the 1b light intermediate chain of flagellar dynein protein. In transformant 4–15 the insertion took place within the promoter region of the gene encoding one short chain dehydrogenase. Curiously, in transformant 3–4 the marker gene was located in the second exon of the ribulose biphosphate carboxylase small chain whose promoter is, alone or fused to the *HSP70A* promoter, the most widely constitutive promoter used to express foreign genes in *Chlamydomonas reinhardtii* [33]. In transformants 1–14, 2–6, and 3–8 the insertion took place within genes encoding proteins with unknown functions. In transformant 2–9 the *APHVIII* marker gene was inserted in the first exon of a protein predicted as an ubiquitine regulatory protein (UBIRP).

**Table 2:** Molecular analysis of some obtained mutants. Localization of the marker insertion site and identification of the protein encoded by the interrupted gene, when available, are shown. The arrows indicate the site and the sense of the insertion. In the scheme of the insertion site: grey color represents the untranslated region, orange color are the exons and lines the introns, as represented in the *Chlamydomonas* genome database [34].

Mutant	Insertion site	Nearest protein	Scheme of the detailed insertion site
1–14	1:5437700	No functional annotations for this locus	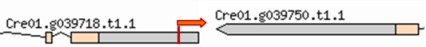
		(Cre01. g039718)	
2–6	3:7536446	No functional annotations for this locus	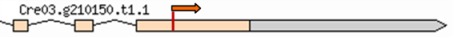
		(Cre03. g210150)	
2–9	3:5019967	Predicted Ubiquitine regulatory protein	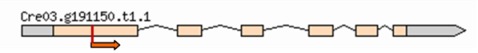
		(Cre03. g191150)	
3–4	2:6042257	Ribulose bisphosphate carboxylase small chain	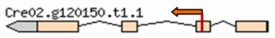
		(Cre02. g120150)	
3–8	11:6509508	No functional annotations for this locus	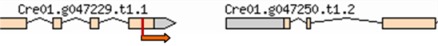
		(Cre01. g047250)	
4–5	2:8218509	Dynein 1b light intermediate chain; D1bLIC	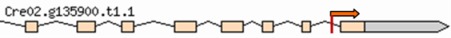
		(Cre02. g135900)	
4–15	2:7024090	Predicted dehydrogenase short chain	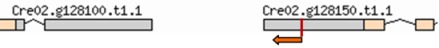
		(Cre02. g128150)	

### 2.4. Testing the Promoter of the 2–9 Transformant

**Figure 3:**
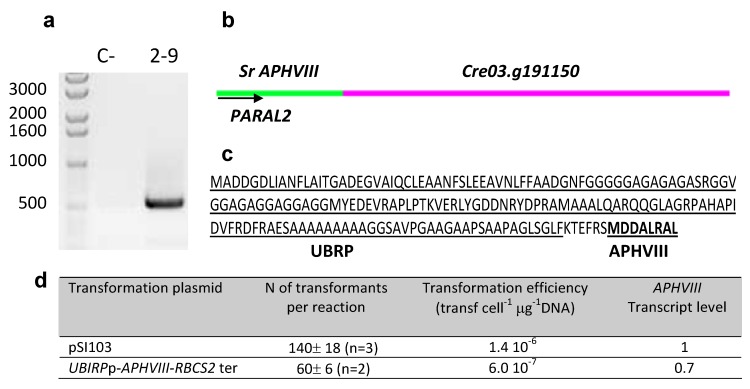
Detailed molecular characterization of transformant 2–9. The genomic DNA fragment amplified during the second round of the iPCR preformed in 2–9 transformant was separated by agarose gel electrophoresis (**a**). The product was sequenced and analyzed using the BLAST tools of the NCBI and the *Chlamydomonas* genome data (**b**). The amino acid sequence translated from the amplified sequence shows that the predicted UBIRP and APHVIII proteins are in the same reading frame (**c**). The efficiency of the nuclear transformations and the relative fold difference between the *APHVIII* transcript level in transformants of *Chlamydomonas reinhardtii* obtained with *UBIRP-APHVIII* and with pSI103 plasmid (**d**) are also shown.

The genomic region preceding the marker gene in transformants that exhibit high antibiotic tolerance should drive the expression of foreign genes with high efficiency. As an example, the 5′ untranslated region isolated from the transformant 2–9, which corresponds to the promoter region of protein Cre03.g191150, predicted as an ubiquitine regulatory protein (UBIRP), was used to drive the expression of the marker *APHVIII* gene in *C. reinhardtii*. Details about the marker insertion site in this transformant are shown in Figure 3. The second round of the inverse PCR allowed the isolation of a DNA fragment of about 500 bp. The first 30 nucleotides of this sequence corresponded to the *APHVIII* marker, confirming the specificity of the amplification. The following nucleotides of the fragment had 100% homology with the protein Cre03.g191150 of chromosome 3 as shown in Figure 3. Detailed analysis of the sequence confirmed that the *APHVIII* marker gene is in the same reading frame that the predicted ubiquitine regulatory protein.

The promoter of the ubiquitine regulatory protein was amplified by PCR with the primers UBIRP-F and UBIRP-R (Table 3) and fused to the *APHVIII* gene by inserting it among the *Not*I/*Bst*BI restriction sites of pSI104-PLK plasmid [35]. The obtained plasmid was used for transformation of *Chlamydomonas reinhardtii* by the glass beads method as described in materials and methods. The pSI103 [30] plasmid in which the *APHVIII *gene is under the control of *HSP70A/RBCS2* promoter was used for comparison. The efficiency of the new identified promoter was high, reaching 60 transformants per reaction and per µg of DNA (which means a transformation efficiency of about 6.0 × 10^−7^ transformants per μg DNA cell^−1^), though it was lower than that observed for the plasmid pSI103 which was about 1.4 × 10^−6^, with 140 transformants per µg DNA (Figure 3). In this case, about 1 µg of plasmid DNA was used for each transformation. 

**Table 3:** Nucleotide sequences of primer pairs used for PCR amplifications.

Primer	Sequence (5′→3′)	Uses
APH8-F*BAM*	CGCCCTCCCCGGATCCGAAGAA	Amplification of the *APHVIII* gene from pSI103 plasmid
APH8-R*SAC*	ACCCACGAGCTCCAACCCTACCC	
NRfor	GCGCTGCCCTCCGTCACCTTCC	Estimation of the number of *NR* genes by qPCR
NRre	CAGCCGCACGCCCGTCCAGTAG	
Parafor	GAGGATCTGGACGAGGAGCGGAA	Estimation of the number of *APHVIII* genes and the *APHVIII *transcript level by qPCR
Pararev	CCCTCAGAAGAACTCGTCCAACAGC	
qUbqL-for	GTACAGCGGCGGCTAGAGGCAC	Houskeeping gene for estimation of the *APHVIII* transcript level
qUbqL-rev	AGCGTCAGCGGCGGTTGCAGGTATCT	
ParaR 1	GTGGAGGGTGGTGGGGACGAGAGG	Identification of the region upstream the marker gene by iPCR
ParaR 2	GGTGTCCGTTCGATCGCAGTCTC	
ParaL1	GCCCACCACCCCGAAGCCGATAAA	
ParaL2	GGCCCCATCCTCCACAACAA	
UBIRP-F	GCTGCCCGCGACTGTGATGTA	Amplification and subcloning of the promoter identified in mutant 2-9
UBIRP-R	GGGCCGCTGCTGCACCAAACGC	
UBIRP-F*Not*I	GGCGGCCGCGACTGTGATGTA	
UBIRP-R*Bst*BI	GGTTCGAAGCTGCACCAAACGC	

Several random transformants obtained with *URRP-APHVIII* and *HSP70A/RBCS2-APHVIII* constructions were chosen for determination of the number of *APHVIII* gene copies. In three one-copy transformants of each type total mRNA was isolated as described in the experimental section and the *APHVIII* transcript level was calculated on the basis of the relative quantification analytical method (∆∆Ct), using *UBQL* as internal standard. The *APHVIII* transcript level in transformants obtained with the *UBIRP-APHVIII* construction was 0.7 fold the *APHVIII* transcript level found in control transformants obtained with the pSI103 plasmid. The presence of additional regulatory sequences, such as the *HSP70A* promoter and the first intron of the *RBCS2* gene, fused up and downstream the *RBCS2* promoter in the pSI103 plasmid may contribute to the relative higher efficiency of expression as compared to the *UBIRP* alone. Previous studies have demonstrated a synergistic effect of the combined use of the *HSP70A* and *RBCS2* promoters with the *RBCS2* first intron to drive transgene expression in *Chlamydomonas* [30,33].

## 3. Discussion

### 3.1. Choice of the Selectable Marker Gene for Promoter Trapping

In promoter trapping experiments, reporter genes lacking a promoter are randomly inserted into a position of the genome where its transcription relies on its adequate insertion in a region adjacent to an endogenous genomic promoter or in frame with a native gene. This strategy has been widely used in higher plants allowing the screening of differentially regulated genes or monitoring gene expression profiles [21,22,36]. In *Chlamydomonas reinhardtii* the argininosuccinate lyase (*ARG7*) [24] or the radial spoke protein (*RSP3*) [23] genes were successfully used for promoter trapping, on the basis of restoring the ability to grow in the absence of arginine or the motility in *arg7* and *pf-14* mutants, respectively. Other marker genes, such as versions of *GFP* [37] or luciferase [38] adapted to *Chlamydomonas* nuclear codon usage, or the arylsulphatase [25] are available for *Chlamydomonas* and have been successfully used for easy assay of promoters activity. However, the most powerful selectable markers are those conferring resistance against antibiotics such as bleomycin (*BLE*) and paromomycin (*APHVIII*). The *APHVIII *gene has been widely used for transformation of *Chlamydomonas*, making possible optimization of promoters [30] and preparation of knockout insertional mutants [26] but, to date, no promoter trapping system based on this marker has been described. 

Here, we show that the selection of vigorous transformants under increasing antibiotic concentrations allows the identification of strong constitutive promoters. We chose *APHVIII* as marker gene because it provides a stable phenotype, the antibiotic paromomycin lacks the mutagenic activity described for other antibiotics, such as bleomycin [29]. It is an exogenous gene easy to identify by PCR, and selection of transformants does not rely on restoring phenotypes of mutant hosts, such as the systems based on *RSP*3 or *ARG7* genes. Furthermore, certain frequency of transformation has already been reported with promoter-less *APHVIII* genes in *Chlamydomonas* [30]. The use of an exogenous promoter avoids the possibility of homologous recombination, which has been reported to happen at low frequency (0.01%–0.7%) in *Chlamydomonas* [23]. We observed that in 100% of the transformants the insertion took place in intragenic regions, while in promoter trapping experiments preformed in higher plants, a high percentage of insertions were reported to occur in intergenic regions [36,39]. The authors explain reporter expression in these transformants from cryptic promoter activity in such regions or from the promoter activity of unannotated genes.

To identify the promoter DNA in each of the transformants chosen, we used an inverse nested PCR over digested and relegated genomic DNA (Figure 2). Other strategies widely used for this purpose in higher plants or microalgae include: Thermal asymmetric interlaced PCR [40], adaptor ligation-mediated PCR [36], rapid amplification of 5′ complementary DNA ends (5′ RACE) [23], or RESDA PCR [41]. Tonooka and Fujishma [42] have recently offered a critical review of these methods to walk along genomes. Similar inverse PCR strategy has been successfully used for identification of the regions flanking the *APHVIII* gene in insertional mutants of *Chlamydomonas reinhardtii* [43]. 

### 3.2. Application to Other Microalgal Species

Strong constitutive promoters, which drive the expression of abundant proteins, such as the RBCS2 [33] or the PSAD [44] in *Chlamydomonas* or the fucoxanthin chlorophyll binding protein (FCP) in *Phaeodactylum* [3] have been widely used to drive the expression of heterologous proteins in these microalgae. However, the promoters of the corresponding genes of other species are not necessarily as efficient. Walker and coworkers *et al.* [12] isolated two *RBCS* genes in *Dunaliella tertiolecta* and used their promoters to drive the expression of the gene encoding bleomycin resistance (*BLE*) in the own *D. tertiolecta*. They only recovered one stably transformed *Dunaliella* line [16]. Therefore, a wise selection of strong promoters based on marker genes should be necessary to identify the strongest promoters in each species. In preliminary experiments, not shown here, we have determined the lethal doses of paromomycin for other chlorophytes, showing that this antibiotic can be a useful selective agent for many freshwater green microalgae and thus the *APHVIII* gene could be used as marker gene in many other microalgae. Following the strategy described here, *APHVIII*-based systems could be designed for promoter trapping in other species.

Identification of promoters once the region preceding the marker has been sequenced is not always an easy task. Eukaryotic promoters are typically located upstream of the gene open reading frame and most of them contain the sequence TATAAA (the TATA box) which binds a TATA binding protein, and assists RNA polymerase to initiate transcription. However, this characteristic element is not always present in all promoters and often other regulatory elements far away from the transcriptional start site can be important to enhance promoter activity. Furthermore, the marker gene can be expressed not only when inserted following a promoter region, but also when inserted in frame with a native gene. The availability of the whole genome sequence and a large number of annotated proteins in *Chlamydomonas* is a great advantage, which has made possible a detailed study of the insertion site in several of the paromomycin-resistant transformant obtained. In other microalgal strains, for which the genome sequence is not available, it should be necessary to isolate several transformants and check the ability of the region preceding the marker gene to act as a promoter in all of them, to have higher probabilities to isolate a real promoter. However, the growing number of microalgal genomes sequenced will surely facilitate the identification of promoters in these species. Beside *Chlamydomonas* [45] and *Phaeodactylum* [46], the nuclear genome sequencing project of other microalgae such as the small *Ostreococcus tauri* [47], *Micromonas* [48] and more recently *Nannochloropsis gaditana *[20], have been completed, and many other are in progress [49].

## 4. Experimental Section

### 4.1. Microorganisms and Culture Conditions

*Chlamydomonas reinhardtii* cell-wall deficient strain 704 (*cw15*, *arg7*, mt^+^) was kindly provided by Dr. Roland Loppes [50] and cultured photomixotrophically in liquid or agar solidified TAP medium [51] at 25 °C under continuous white light irradiation of 100 µE m^−2^ s^−1^. The *Escherichia coli* strain used for *in vivo* amplification of DNA was DH5α, cultured in LB medium as previously described [52].

### 4.2. Nuclear Transformation of *Chlamydomonas reinhardtii* with the *APHVIII* Promoter-Less Cassette

Transformants for promoter trapping were generated by transformation of the *Chlamydomonas reinhardtii* strain 704 (*cw15*, *arg7*, mt^+^) with a DNA cassette containing the aminoglycoside 3′-phosphotransferase gene (*APHVIII*) from *Streptomyces rimosus*, followed by the nopaline synthase (*NOS*) terminator region from *Agrobacterium tumefaciens*. 

Transformation was carried out using the glass-bead method of Kindle [53], with minor modifications. *C. reihardtii* cells were grown until the middle of the exponential phase of growth (about 1.6 × 10^6^ cells mL^−1^), harvested by centrifugation and resuspended in fresh TAP medium to obtain a 100 fold concentrated cell suspension. The concentrated cell suspension (0.6 mL) was added to a conical tube containing 0.3 g of sterile glass beads (0.4–0.6 mm diameter), 0.2 mL of 20% polyethylene glycol (MW8000) and the indicated quantities of the chosen cassette or plasmid. Cells were vortexed for 8 s and resuspended in 50 mL of fresh sterile TAP medium where they were incubated overnight. After this incubation in the absence of antibiotic, the cells were pelleted and spread onto TAP solid medium plates with paromomycin (30 µg mL^−1^). Transformed colonies were visible after 4 or 5 days.

### 4.3. Isolation of Genomic DNA

The pellet obtained after centrifugation of 2 mL of *Chlamydomonas* culture was resuspended in 300 µL of lysis buffer (50 mM Tris-HCl, pH 8.0, 5 mM EDTA, 2% SDS, 0.3 M NaCl), vigorously vortexed for 10 min and incubated in ice for 2 min. DNA was extracted with phenol/chloroform and precipitated with absolute ethanol, overnight at −20 °C. The pellet was washed with ethanol 70%, dried and resuspended in 40 µL of 5 mM Tris-HCl, pH 8 [36].

### 4.4. RNA Extraction and Reverse Transcription

Isolation of total RNA was performed with the RNAeasy plant MiniKit of Qiagen according to instructions of the manufacturer. Single strand cDNA was synthesized from total RNA according to the SuperScript II RNaseH-reverse transcriptase manual (Invitrogen) and used as substrate for Real Time-PCR reactions. 

### 4.5. Standard Polymerase Chain Reaction

The PCR amplification was performed from 1 µL of genomic DNA in a total volume of 25 µL containing 10 pmol of each primer, 0.2 mM dNTPs, 0.5 U *Taq* DNA polymerase from Biotools (B & M Labs, Madrid, Spain), 2.5 μL of specific 10× buffer (containing 2.5 mM MgCl_2_), and 1% dimethylsulfoxide (DMSO). The PCR program was: 0.5 min at 96 °C, 0.5 min at annealing temperature, and 1.5 min at 72 °C for 30 cycles.

### 4.6. Inverse PCR

Inverse PCR was preformed by digesting 500 ng of RNAase treated genomic DNA with the restriction endonuclease *Sal*I, which has a unique restriction site inside the *APHVIII-NOS *cassette and has a high number of target sites in the genome of *Chlamydomonas*. The resulting digestions were precipitated by adding ethanol 95% and sodium acetate at a final concentration of 100 mM and resuspended in 8 µL of water. The obtained digestion products were ligated with T4 ligase overnight at 16 °C and used as a template for nested PCR (Figure 2) using different pairs of inverted primers (Table 3). Each PCR reaction was carried out at the standard conditions described before, excepting that cycling conditions were 1× (96 °C, 5 min), 35× (95 °C, 1 min; 60 °C, 1 min; 72 °C, 3 min), 1× (72 °C, 10 min).

### 4.7. Analysis of the Insertion Sites

The products amplified by inverse PCR were separated by agarose gel electrophoresis. The fragments obtained were isolated with a gel extraction kit (Qiagen) and sequenced. The resulting sequences were analyzed by comparison with *Chlamydomonas* genome database [34].

### 4.8. Quantitative Real-Time PCR. Transcript Level Analysis and Determination of the Number of Copies of the *APHVIII* Gene Integrated in the Genome of *Chlamydomonas*

Real time PCR was performed on a Mx3000P Multiplex Quantitative PCR System from Stratagene using the Brilliant SYBR Green QPCR Master Mix (Agilent). Each determination was carried out in triplicate using genomic DNA as template, and 10 pmoles of the indicated primers in a final volume of 25 µL. Cycling conditions were: 10 min at 95 °C for activation of the hot start Taq polymerase and 40 cycles for the melting (30 s at 95 °C), annealing (30 s at 60 °C) and extension (30 s at 72 °C). The fluorescence measurement was made at the end of the annealing step. A dissociation curve (30 s at 95 °C, 30 s at 55 °C and 30 s at 95 °C) was applied at the end of the amplification reaction to check possible formation of dimmers.

For transcript level analysis, the ubiquitine ligase gene (*UBQL*, GenBank BU648530), which encodes the ubiquitine ligase protein, was used as housekeeping gene. Expression of this gene was previously checked to be constitutive under the different conditions used [41]. Primers efficiencies were 1.04 and 1.00 for *APHVIII* and *UBQL*, and yield amplicons 359 bp of 161 bp, respectively.

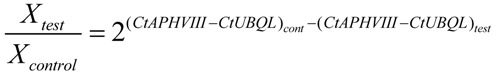
(2)


For determination of the number of *APHVIII* copies, nitrate reductase (*NIA1*) was used as one copy gene reference. Using specific primers with very similar efficiencies for *APHVIII* and *NIA1* (Table 3) the number of integrations was calculated using the ∆∆Ct method [54]. Efficiencies of the primers were previously determined [26]. 

## 5. Conclusions

Promoter trapping, which has been widely used for the selection of regulable promoters induced under certain nutritional or environmental conditions, can also allow the selection of strong endogenous promoters. We have demonstrated that transformation with the promoterless *APHVIII* gene from *Streptomyces rimosus*, which encodes for an aminoglycoside 3′-phosphotransferase and provides resistance to the antibiotic paromomycin, and subsequent isolation of the most robust transformants enables easy identification of novel promoter sequences in *Chlamydomonas*. This strategy could be applied to many chlorophytes sensitive to paromomycin.
